# Fiber-Reinforced Polymer Nanocomposites

**DOI:** 10.3390/nano12173045

**Published:** 2022-09-02

**Authors:** R. A. Ilyas, N. M. Nurazzi, M. N. F. Norrrahim

**Affiliations:** 1 School of Chemical and Energy Engineering, Faculty of Engineering, Universiti Teknologi Malaysia (UTM), Johor Bahru 81310, Malaysia; 2 Centre for Advanced Composite Materials, Universiti Teknologi Malaysia (UTM), Johor Bahru 81310, Malaysia; 3 Institute of Tropical Forest and Forest Products (INTROP), Universiti Putra Malaysia, UPM, Serdang 43400, Malaysia; 4 Bioresource Technology Division, School of Industrial Technology, Universiti Sains Malaysia, Gelugor 11800, Malaysia; 5 Research Centre for Chemical Defence, Universiti Pertahanan Nasional Malaysia (UPNM), Kuala Lumpur 57000, Malaysia

“Fiber-Reinforced Polymer Nanocomposites” is a newly open Special Issue of *Nanomaterials*, which aims to publish original and review papers on new scientific and applied research and make boundless contributions to the finding and understanding of the reinforcing effects of various nanomaterials on the performance of polymer nanocomposites. This Special Issue also covers the fundamentals, characterization, and applications of fiber-reinforced polymer nanocomposites.

Today, nanomaterials are used in several applications, including composites, packaging, electronic, electrical, structural, energy storage, automotive, filtering, and coating applications, among other (Figure 1) [1,2,3,4,5]. The continuous development and appearance on the market of new high-performance reinforcing nanomaterials in polymer composites has constituted a strong challenge for researchers to design and adapt new functional nanocomposites for several applications [2,6,7]. This Special Issue aims to provide deep insights into the reinforcing effects of various nanomaterials on the mechanical, physical, thermal, and electrical performance of polymer nanocomposites. 

Nanomaterials can be classified into natural and synthetic. Nanocellulose, nano-clay, graphene and MXene, carbon nanofibers and nanotubes, silica nanoparticles, and ZnO quantum dots are common nanomaterials used in polymer nanocomposites [8,9,10]. Most have several valuable features, such as being renewable and having large specific surface areas, high crystallinities, and surface functionalization capabilities. Nanomaterials can play two essential roles in polymer nanocomposites. The first is to improve the various performances of the material, such as mechanical, barrier, thermal, flame retardancy, and electrical performances. Meanwhile, the second is the modification of miscibility and morphology of the polymer nanocomposites.

Interestingly, research in the field of fiber-reinforced polymer nanocomposites received a lot of findings that positively contributed to many applications such as biomedical, automotive, electronics, structural materials, packaging [11,12,13,14,15,16,17,18,19], textile, military, gas sensing [20], membrane [21], aerospace [22], heat transfer fluid, and cooling applications [23]. Despite the outstanding achievements obtained thus far, the performance of fiber-reinforced polymer nanocomposites overall is sometimes insufficient for emerging industrial applications. Thus, more studies on the performances such as electrical, thermal, fire-resistant, and electromagnetic shielding are urgently needed. In addition, the nanomaterials market is still far from reaching its full potential. Several challenges exist, including a lack of process-adapted, continuous resources and the measuring tools capable of characterizing nanomaterials to meet industrial demands, in addition to scant expertise and cost inefficiencies.

This Special Issue will cover recent advances in the three primary aspects of processing, characterization, and performance. Both synthetic and natural nanomaterials-based composites are welcome. Moreover, this issue is welcomed in several vital aspects, such as the production of nanomaterials, surface and interfacial characterization of its properties, economic feasibility, challenges, and future perspectives in the field of polymer nanocomposites; as a result, current and future literature data can be enriched.

## Figures and Tables

**Figure 1:**
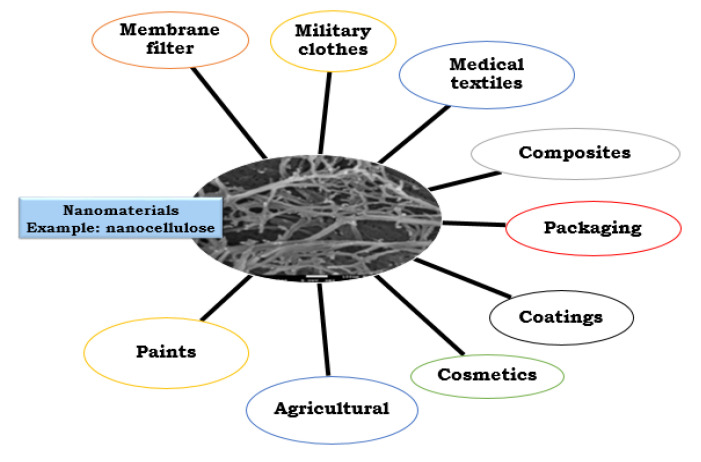
Various applications of nanomaterials.
